# Molecular cloning, spatial and temporal expression analysis of CatSper genes in the Chinese Meishan pigs

**DOI:** 10.1186/1477-7827-9-132

**Published:** 2011-10-04

**Authors:** Chengyi Song, Bo Gao, Han Wu, Yuxiu Xie, Xiaoyan Wang, Bichun Li, Guohong Chen, Jiude Mao

**Affiliations:** 1 College of Animal Science & Technology, Yangzhou University, Yangzhou, Jiangsu, 225009, China; 2 Department of Animal Sciences, University of Missouri, Columbia, MO, 65211, USA

## Abstract

**Background:**

Sperm ion channel proteins (CatSpers) are essential for sperm hyperactivated motility, and then penetration through the zona pellucida. The CatSper class of proteins have well been characterized in the mouse and human. However, such data for pigs are not available. In the present study, we cloned the porcine CatSper 1-4 genes, analysed their spatial expression in various organs and temporal expression in the testes from birth until sexual maturity in Meishan boars.

**Methods:**

Rapid amplification of cDNA ends (RACE) was performed to clone the full length cDNAs of porcine CatSper genes and bioinformatics analysis of inferred CatSper proteins was also determined. Various organs were collected from 150 day-old pigs to characterize the spatial expression of CatSper genes by qualitative reverse transcriptase polymerase chain reaction (RT-PCR), and testes from birth to 150 day-old boars were sampled to detect the temporal expression of CatSper genes by quantitative real-time RT-PCR.

**Results:**

The mRNA sequences of CatSper1 (2452 bp), CatSper2 (2038 bp), CatSper3 (1408 bp), and CatSper4 (1799 bp), including full length of cDNAs, 5' and 3' flanks, were obtained. The bioinformatics analysis indicated that coding regions spanning the ion transport domains were conserved for different species analyzed. Among the four CatSpers, CatSper2, 3, and 4 were more conserved across species, compared with CatSper1. In addition, six conservative trans-membrane domains, a pore forming motif, and a coiled-coil motif were also identified. The spatial analysis from different organs showed that CatSper1 was detected in both testes and hypothalamus, CatSper2 was restricted in testes only, CatSper4 was expressed in testes and rete testes; whereas CatSper3 was more ubiquitously. CatSper3 and CatSper4 transcripts were also detected in ejaculated sperm. At Days 1 and 30 of age, CatSper mRNAs exhibited only sparse expression in the testes. However, these transcripts highly expressed at Day 60 and onward till sexual maturity (Day 150 of age).

**Conclusions:**

The spatial and temporal expression profiles of CatSper genes were reported herein for the first time in pigs. CatSper1, CatSper2 and CatSper4 were primarily expressed in testes, while CatSper3 transcript was prevalent in a variety of organs. CatSper3 and CatSper4 mRNAs were present in mature sperm cells. Substantial upregulation of CatSper genes was initiated at Day 60 and maintained this marked production until sexual maturity.

## Background

The CatSper ion channel proteins were first discovered by Ren et al. [1]. Later it was discovered that this ion channel is a sophisticated protein complex composed of at least six subunits, of which four alpha subunits (CatSper 1-4) form a calcium selective pore [2,3], and two additional auxiliary subunits, CatSperβ and CatSperγ, which are transmembrane proteins with large extracellular domains [4,5]. CatSper1 and CatSper2 were identified to be essential for sperm motility and male fertility in the mouse [6-9]. It was also found that CatSper1 is required for the Ca^2+ ^current activation by alkalinization during sperm cell capacitation [10]; whereas CatSper3 and CatSper4 are essential for sperm hyperactivated motility and male fertility [3,11,12]. Studies with CatSper1, 2, 3, and 4 knock-out mice revealed that all four subunits are required for stable expression of the heteromeric channel complex, and knockout of any one of these subunits result in absence of the other subunits [3,6,12].

In the mouse, the expression of CatSper1-4, CatSperβ and CatSperγ is confined to the testes [1,3-5,11,13]. It was also found that CatSper1, CatSper2, CatSperβ and CatSperγ were localized specifically at the principal piece of mature sperm [1,4,5,13], and CatSper3 and CatSper4 were present in the acrosome of sperm [12]. The mRNA expression of mouse CatSper is developmentally timed to coincide with the appearance of round spermatids [14,15]. Furthermore, the relative CatSper gene expression level is positively correlated with sperm motility in human [14,16].

Porcine represents a classic animal model in biomedical research and an important farm animal in agriculture. However, no data on CatSper genes expression and sequence is currently available. The onset of spermatogensis in Chinese Meishan boars occurs at much earlier age compared with western breed of pigs. the former breed reach puberty at about 75 days, undergo sexual maturity at around 120 days of age [17], and are well known for their extremely high prolificacy with litter size of 15 to 16. Thus, Meishan pigs offer a unique model to characterize factors influencing male reproductive function. As an initial step toward understanding the function of the CatSper proteins in pigs, the present study cloned and sequenced CatSper cDNAs and characterized their temporal expression profiles in the testes during postnatal development, and spatial expression in diverse non-reproductive and reproductive organs, and in ejaculated sperm of Chinese Meishan pigs.

## Methods

### Tissue and sperm sample collection

Animal care and use was approved by the University of Yangzhou University Animal Care and Use Committee. To study spatial expression in different organs, fourteen different organs samples, including brain (frontal lobe), cerebellum, pituitary, hypothalamus, hippocampus, heart, liver, spleen, lung, kidney, colon, small intestine, longissimus muscle, abdominal fat, and twelve reproductive organs, including testes, rete testes, caput, corpus and cauda epididymidis, bulbourethral gland, prostate gland, seminal vesicle, vas deferens, penis from 150-day old boars, ovary and uterus from 150-day old gilts, and sperm samples from three adult Meishan boars at different ages were used for evaluation of CatSper genes. To study temporal expression of CatSper genes during development, testis samples at post-natal days 1 (n = 5), 30 (n = 4), 60 (n = 3), 90 (n = 3) and 150 (n = 3) of age were collected. Organ samples were collected immediately after slaughter, snap-frozen in liquid nitrogen, and stored at -80°C freezer until RNA isolation. Sperm samples were collected and subjected to swim-up in Tyrode's complete medium (TCM) [18] at a 45° angle, 5% CO_2 _and 37°C for 60 min to remove any potential somatic cell contamination. Top fraction (1/2) of sperm samples were separated by centrifugation at 1200 g for 5 minutes at 25°C and washed twice in TCM, and then used for RNA extraction.

### RNA isolation and cDNA Synthesis

Trizol reagent was used to isolate total RNA from the homogenized organ (about 50 mg/ml Trizol) and sperm samples (about 5 × 10^9 ^cells/ml Trizol) according to the manufacturer's protocol (Invitrogen). The first strand cDNA was synthesized by incubating 2 ug of total RNA with Moloney Murine Leukemia Virus reverse transcriptase (M-MLV, Promega, USA) at 42°C for 50 min.

### Molecular cloning of porcine CatSper cDNAs

The full-length cDNA sequences of porcine CatSper genes were obtained by using rapid amplification of cDNA ends (RACE) method. Initially, primers of CatSpers (CatSper1, forward:5'ggttgtgggaaaaattaagc3', reverse:5'ggtagatgagggaccagtcg3'; CatSper2, forward: 5'gatactttctctctcatcgagc3', reverse:5'cattccaggcattcttccag3'; CatSper4, forward: 5'gaggagcaagtgctcatcaa3', reverse:5'gcagaagttgtcgcatgtgt3') were designed according to the homologous regions of CatSper mRNAs from human, cattle, dog, and pig, which are available in the NCBI database. Because of their prolonged coding sequence, only partial fragments of CatSper1, CatSper2 and CatSper4 genes were amplified; whereas, CatSper3 is possesses a short and full length of cDNA product facilitating a full length sequence product with RACE. The PCR procedure was carried out as follows: denatured at 94°C for 3 min, followed by 35 cycles of 94°C 30 sec, 60°C 30 sec and 72°C 60 sec, and final extension at 72°C for 5 min. The PCR products of CatSper1, CatSper2 and CatSper4 were inserted into the pMD18-T TA clone vector (TaKaRa) and sequenced. Based on the cloned sequences and porcine CatSper nucleotide sequence already available in the NCBI database, gene specific primers (refer to Table 1) for CatSper genes were further designed and used to amplify the 5' and 3' ends of CatSper cDNAs by using GeneRacer™ kit (Invitrogen). The final PCR products were cloned into pMD18-T TA clone vector (TaKaRa) and sequenced. The sequenced fragments were assembled to construct the full length of CatSper cDNAs. The resulting sequences were analyzed for similarity with other species by using the on-line BLAST program [19].

**Table 1:** Primers of CatSper genes used in 5' and 3'RACE

Gene	5'RACE		3'RACE	
	Primers	Sequence 5'-3'	Primers	Sequence 5'-3'
*CatSper1*	GSP1	aagtaccactcgccccgga	GSP1	cctgttctccgtggtgctc
	GSP2	gacctcggcgaaggtctg	GSP2	gagcatcctcaccaccatct
	GSP3	gcagatgatgaagacgatgaagg		
*CatSper2*	GSP1	atccgtcctacgtgacaagg	GSP1	tgaagctgactctggaggtg
	GSP2	gcacaagctggtgttgatct	GSP2	tgttttcatcttggagatccttc
	GSP3	gtatctctcggatggtgtgc		
*CatSper3*	GSP1	gctgtaggtgataagcttgagga	GSP1	ctactggagggacggctaca
	GSP2	gtcggcaatgttgaggtagg	GSP2	catcatccttgccatcctct
	GSP3	tagtgcttgcccttgacctt		
*CatSper4*	GSP1	ccccaggagcaagataaaga	GSP1	cactggtgcattgtgtggtc
	GSP2	cagataaggatggtcagcacaa	GSP2	acctctcggaaaacacatgc
	GSP3	tcagcacaatgtcatctatgg		

### Bioinformatics analysis

Based on the CatSper gene sequence, the amino acid orthologous sequences were deduced. The deduced porcine amino acid sequences and the ones for human, mouse, rat, cattle, and dog from NCBI database were aligned by using the CLUSTALW2 program [20]. The transmembrane domains in the protein alignment of CatSpers were predicted by tmap program [21]. The coiled coil motifs in porcine CatSper proteins were analyzed by COILS program [22].

### RT-PCR assay

The qualitative reverse transcription polymerase chain reaction (RT-PCR) was used to detect the spatial expression of CatSper genes, and the quantitative real-time RT-PCR was used to examine the temporal expression of CatSper genes during testicular development. Primers (list in Table 2) were redesigned based on sequences of cloned CatSper cDNAs and PCR protocols were summarized in Table 2. Porcine glyceraldehyde-3-phosphate dehydrogenase (GAPDH), a housekeeping gene, was used as a positive control to confirm the presence of cDNA. Thirty and 35 cycles were applied for GAPDH and CatSper gene amplifications in organ samples, respectively. To detect the expression of CatSper genes in sperm, 42 cycles were applied and the purity of sperm RNAs was determined by RT-PCR with three sets of primers (list in Table 2) amplifying c-kit, CD4 and E-Cadherin cDNA, which are positive marker for testicular germ cells, leukocytes and epithelial cells, respectively [16,23]. The quantitative real-time RT-PCR was performed on an ABI 7500 Fast Real-Time PCR System (ABI) with SYBR^® ^Premix Ex Taq™II (TaKaRa) according to the protocol summarized in Table 2. The threshold cycles (C_t_) of CatSper genes were normalized with GAPDH. Relative quantification of the expression of genes was calculated using the 2^-ΔΔCt ^method [24].

**Table 2:** Porcine primer sequences and PCR protocols for the genes used in RT-PCR analysis.

Primers	Sequence 5'-3'		Product size (bp)	PCR protocol				
	Forward	Reverse		94°C	94°C	60°C	72°C	72°C
*CatSper1^a^*	gagcatcctcaccaccatct	gtgttcgctcatcacctcct	310	3 min	30 sec	30 sec	30 sec	5 min
	ggtgactcccacctctttga	cttcttttctggctcgatgg	111	3 min	30 sec	30 sec	20 sec	5 min
*CatSper2^a^*	tgttttcatcttggagatccttc	gtagacaccagccacagcaa	319	3 min	30 sec	30 sec	30 sec	5 min
	cttgcacgattccctcaaat	gcgagttgaacgggtgtaat	153	3 min	30 sec	30 sec	20 sec	5 min
*CatSper3^a^*	ctactggagggacggctaca	gccaagctgaagagggtaaa	348	3 min	30 sec	30 sec	40 sec	5 min
	gctggtgcagacacagaaaa	atcctggttgtccagagtgg	160	3 min	30 sec	30 sec	20 sec	5 min
*CatSper4^a^*	acctctcggaaacacatgc	ggctgggtgtgtttgcttat	350	3 min	30 sec	30 sec	35 sec	5 min
	ggacacccgtgagatgactt	ggctgggtgtgtttgcttat	157	3 min	30 sec	30 sec	20 sec	5 min
*GAPDH^a^*	acccagaagactgtggatgg	ccccagcatcaaaggtagaa	346	3 min	30 sec	30 sec	35 sec	5 min
	catggcctccaaggagtaag	tctgggatggaaactggaag	150	3 min	30 sec	30 sec	20 sec	5 min
*CD4*	tggaaacctgaccttggttc	agaacccagcgagaaacaga	359	3 min	30 sec	30 sec	35 sec	5 min
*E-Cadherin*	ccccatgttcgaatatccac	tgctgtgacaagccatcttc	396	3 min	30 sec	30 sec	40 sec	5 min
*c-kit*	ttgttgatgacctcgtggaa	atggaatctgaggccttcct	307	3 min	30 sec	30 sec	30 sec	5 min

^a^First primer pairs were used for the qualitative RT-PCR, and second primer pairs were used for the quantitative real-time RT-PCR.

### Statistical analysis

The expression level of CatSper genes in testes were calculated at each time point. Data were analyzed by using SPSS16 for Windows Software and presented as Mean ± SEM. Differences of gene expression level between ages were analyzed by ANOVA, followed by post hoc tests by using the LSD test and the statistical significance was accepted when P was less than 0.05 (compared with their former time point).

## Results

### Molecular cloning of porcine CatSper cDNAs

**Figure 1:**
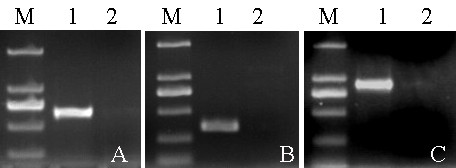
**Fragments of CatSper1, CatSper2, and CatSper4 cDNAs**. A. approximately 650 bp of CatSper1; B. 450 bp of CatSper2; C. 900 bp of CatSper4, amplified by using primers designed according to the available CatSper nucleotide sequence of pig and the conserved sequences of CatSpers among animals. Lane 1 is the PCR products from testis cDNAs, lane 2 is the negative control, lane M is the DL2000 DNA marker.

The available porcine CatSpers cDNA sequences from the NCBI database [25] included 125 bp for CatSper1 [GenBank:AM947645], 137 bp for CatSper2 [GenBank:AM947646], 169 bp for CatSper3 [GenBank:AM947647] and 174 bp for CatSper4 [GenBank:AM947648]. To facilitate the 5'RACE, three sets of primers correspond to CatSper1, CatSper2 and CatSper4 cDNAs were designed, according to the available CatSper nucleotide sequences of pig and the conserved sequences of CatSpers among other mammals. Three PCR products of CatSper1, CatSper2 and CatSper4 cDNAs with expected size of 650 bp, 450 bp, and 900 bp were obtained (Figure 1). Then, the purified cDNA products were inserted into TA clone vectors and sequenced. The sequencing results were confirmed by analyzing for similarity with known CatSper sequences of other species using the BLASTN program. The mRNA sequences, including the 5' and 3' un-translated regions and full-length cDNAs of porcine CatSper1, CatSper2, CatSper3 and CatSper4 genes, were successfully obtained by RACE method. These sequences have been deposited into GenBank with accession number of HQ654519, HQ654520, HQ654521, and HQ654522. The characteristics of porcine CatSper mRNA sequences were predicted by DNAStar and are summarized in Table 3. The lengths of cloned mRNA sequences of CatSper1, CatSper2, CatSper3 and CatSper4 genes were 2452, 2038, 1408, and 1799 bp, respectively. They contain 2169, 1599, 1203, and 1350 bp open reading frames (ORFs), respectively, flanked by 5' and 3' terminal un-translated regions (Table 3).

**Table 3:** The characteristics of porcine CatSper mRNA sequences

Gene	mRNA	5' UTR	ORF	3'UTR	Coding Amino acids	MW (kDa)
*CatSper1*	2452	99	2169	184	722	82.8
*CatSper2*	2038	333	1599	106	532	62.1
*CatSper3*	1408	121	1203	84	400	46.5
*CatSper4*	1799	47	1350	402	449	51.8

### Amino acid identity analysis cross animal species

**Figure 2:**
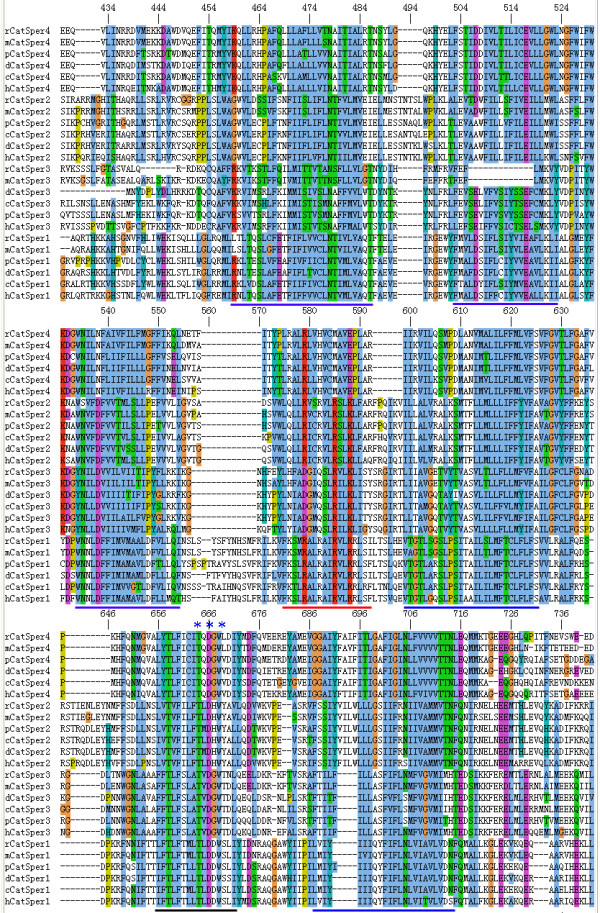
**Multiple sequence alignment of the CatSpers across the ion transport domains**. The human (h), mouse (m), rat (r), dog (d), cow (c), and pig (p) CatSper proteins are aligned (for access number, refer to Table 4), and identical residues are shaded with different color. Predicted transmembrane regions are underlined in blue and the S4 voltage sensor transmembrane helix is underlined in red. The pore-forming region is underlined in black. The conserved residues (T×D×W) in the pore loop were marked with stars.

**Figure 3:**
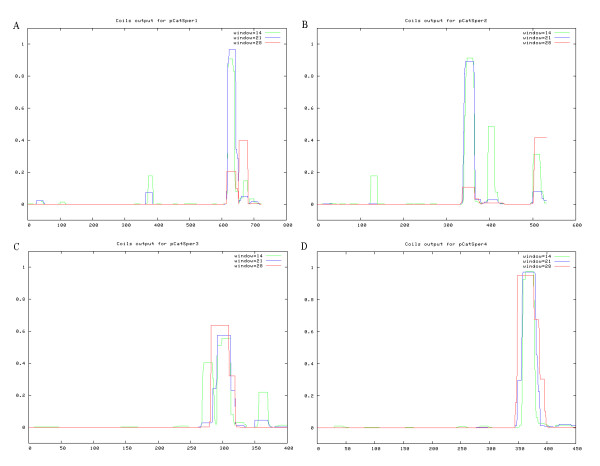
**CatSper1 (A), CatSper2 (B), CatSper3 (C), and CatSper4 (D) channel coiled-coil predictions**. X-axis, amino acid residue numbering of query sequence. Y-axis, probability score for a sequence adopting a coiled-coil configuration calculated for a scanning window of 14, 21 or 28 amino acid residues.

Based on the gene sequences, the amino acid sequences of the four porcine CatSpers were deduced. The amino acid identity between pig and human, mouse, rat, cattle, and dog (access number refer to Table 4) was then analyzed by using CLUSTALW multiple sequence alignment program. The alignment data showed that the regions across the ion transport domains were predictably highly conserved (Figure 2). In contrast low sequence identity in the N and C-terminals, and other regions was observed. Six transmembrane domains (S1-S6) in the protein alignment were predicted by tmap program. A short, conserved hydrophobic stretch representing the pore-forming region is present in a longer loop region between the fifth and sixth transmembrane domains (Figure 2). The sequences in the pore loop represented a similar conserved motif (T×D×W) (Figure 2), which is characterized in voltage-gated calcium channels [26]. Overall, the amino acid sequences of these motifs also appear to be highly conserved across species (Figure 2). A coiled-coil domain at the C terminus of each member of the porcine CatSper family was predicted by COILS program and shown in Figure 3. This domain is well characterized as potential protein-protein interaction motif with various complexes [27].

**Table 4:** Sequence identities shown between CatSper family members

Gene	cCatSper1	dCatSper1	hCatSper1	pCatSper1	mCatSper1	rCatSper1	Access number
cCatSper1	100.0	76.1	63.7	68.1	54.4	54.8	XP_601857
dCatSper1		100.0	70.9	76.3	60.9	62.8	XP_533225
hCatSper1			100.0	57.4	48.7	46.8	NP_444282
pCatSper1				100.0	47.5	75.1	HQ654519
mCatSper1					100.0	45.7	NP_647462
rCatSper1						100.0	XP_001070492
**Gene**	**cCatSper2**	**dCatSper2**	**hCatSper2**	**pCatSper2**	**mCatSper2**	**rCatSper2**	
cCatSper2	100.0	84.5	76.5	87.5	68.8	70.3	NP_001179406
dCatSper2		100.0	79.4	88.5	68.8	69.5	XP_851279
hCatSper2			100.0	79.0	67.6	67.6	NP_473361
pCatSper2				100.0	69.0	77.7	HQ654520
mCatSper2					100.0	70.9	NP_694715
rCatSper2						1	NP_001012220
**Gene**	**cCatSper3**	**dCatSper3**	**hCatSper3**	**pCatSper3**	**mCatSper3**	**rCatSper3**	
cCatSper3	100.0	84.4	70.4	84.0	65.2	64.1	XP_613302
dCatSper3		100.0	74.0	83.0	63.4	60.8	XP_538633
hCatSper3			100.0	69.6	65.7	64.5	NP_821138
pCatSper3				100.0	65.7	85.0	HQ654521
mCatSper3					100.0	62.5	NP_001099571
rCatSper3						100.0	NP_084048
**Gene**	**cCatSper4**	**dCatSper4**	**hCatSper4**	**pCatSper4**	**mCatSper4**	**rCatSper4**	
cCatSper4	100.0	70.3	68.0	76.9	65.2	62.3	XP_599038
dCatSper4		100.0	71.4	80.2	69.2	64.5	XP_544484
hCatSper4			100.0	74.2	68.6	65.3	NP_937770
pCatSper4				100.0	69	85.1	HQ654522
mCatSper4					100.0	68.7	NP_808534
rCatSper4						100.0	XP_342942

human (h), mouse (m), rat (r), dog (d), cow (c), and pig (p)

**Figure 4:**
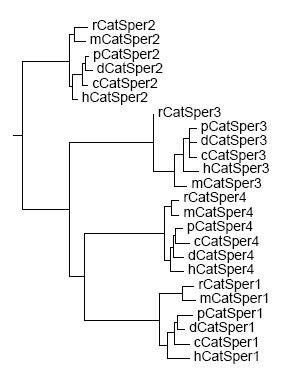
**A phylogenic tree showing the similarity between CatSper protein amino acid sequences across human (h), mouse (m), rat (r), dog (d), cow (c), and pig (p)**. The phylogenic tree was constructed with Neighbor-Joining, and the degree of confidence was estimated by Bootstrap analysis.

The protein sequence identities between CatSper family members were also analyzed and were presented in Table 4. Furthermore, a phylogenic tree was constructed based on the alignment result (Figure 4). Overall, CatSper2, CatSper3, and CatSper4 forms were highly conserved. The sequence identity ranges from 67.6% (mouse vs. human) to 88.5% (pig vs. dog) for CatSper2 orthologs, 60.8% (rat vs. dog) to 85.0% (pig vs. rat) for CatSper3, and 62.3% (rat vs. cattle) to 85.1% (pig vs. rat) for CatSper4 (Table 4). CatSper1 was under a lower evolutionary constraint with sequence identity varying from 45.7% (rat vs. mouse) to 76.3% (pig vs. dog) (Table 4). The phylogenic tree indicated that CatSpers (CatSper1-4) were highly clustered together in dog, pig, and cattle, then with human, and finally with rat and mouse (Figure 4), which is in agreement with the sequence identity distribution for each of them (Table 4). CatSper1 and CatSper4 proteins across species displayed the greatest degree of homology, as evidenced by their tight clustering in gene comparisons, then with CatSper3. CatSper2 protein was clearly distinct from the other members of the family (Figure 4).

### Spatial expression of CatSper genes

**Figure 5:**
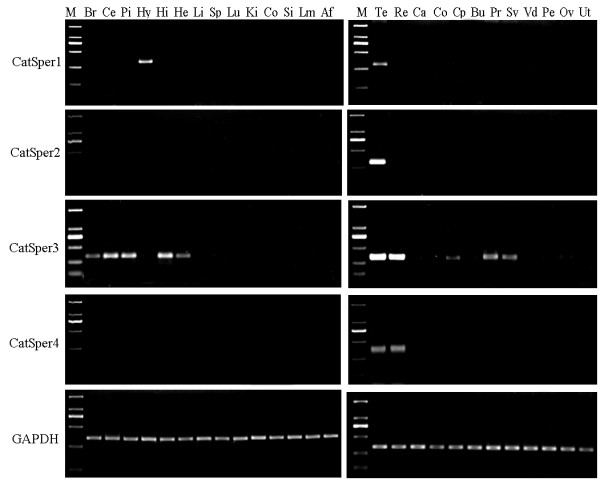
**Spatial expression of CatSpers in non-reproductive (left) and reproductive organs (right) of 150-day old boars and gilts**. Br, brain, Ce, cerebellum, Pi, pituitary, Hy, hypothalamus, Hi, hippocampus, He, heart, Li, liver, Sp, spleen, Lu, lung, Ki, kidney, Co, colon, Si, small intestine, Lm, Longissimus muscle, Af, Abdominal fat, Te, testes, Re, rete testes, Ca, Cauda epididymidis, Co, Corpus epididymidis, Cp, Caput epididymidis, Bu, bulbourethral gland, Pr, prostate, Sv, seminal vesicle, Vd, vas deferens, Pe, penis, Ov, ovary, Ut, uterus.

To determine the spatial expression pattern, complementary DNA was synthesized from 14 non-reproductive organs, 12 reproductive organs, and sperm samples of adult Chinese Meishan pigs. It was found that only the testes expressed the CatSper2 transcript, implying that evolved selectively to support normal testicular function. CatSper1 was only identified in testes and hypothalamus; whereas CatSper4 mRNA was expressed in both testes and rete testes. CatSper3 mRNA was expressed in several organs tested, with strong signal detected in cerebellum, pituitary, hippocampus, testes and rete testes, and faint signal in brain, heart, caput epididymidis, prostate and seminal vesicle (Figure 5). None of these genes was expressed in the ovary and uterus of female pigs. All RT-PCR products of CatSper genes were confirmed by sequencing.

**Figure 6:**
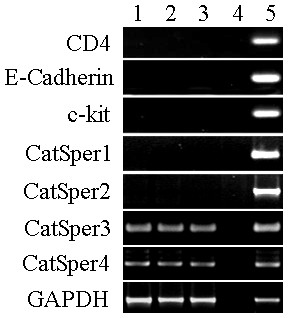
**Expression of CatSpers in the ejaculated sperm**. Lanes 1 to 3: three independent sperm cDNA samples; lane 4: negative control; and lane 5: positive control with cDNA from different tissues (blood leukocytes for CD4, seminal vesicle for E-Cadherin and testes for c-kit and CatSpers).

To determine the transcripts of CatSper genes in ejaculated sperm, the swim-up technique was applied to decrease the potential contamination by somatic cells. Then, the sperm RNA samples were then further assessed for purity and gene expression by using an RT-PCR primers directed against c-kit, CD4, and E-Cadherin makers for germ cells, leukocytes, and principal epithelial cells, respectively [16,23]. Sperm samples without detectable contamination for other cell types were then employed for RT-PCR analysis of CatSper genes. The predicted genes were observed in positive control cDNAs and no signal was detected in negative control cDNA. GAPDH was used as the loading control with detection of this transcript after 35 PCR cycles, while c-kit, CD4, and E-Cadherin was not observed even after 42 cycles (Figure 6). CatSper3 and CatSper4 were detected in sperm with 42 PCR cycles, but the transcripts of CatSper1 and CatSper2 were not detectable within this allotted time (Figure 6).

### Temporal expression of CatSperm genes

**Figure 7:**
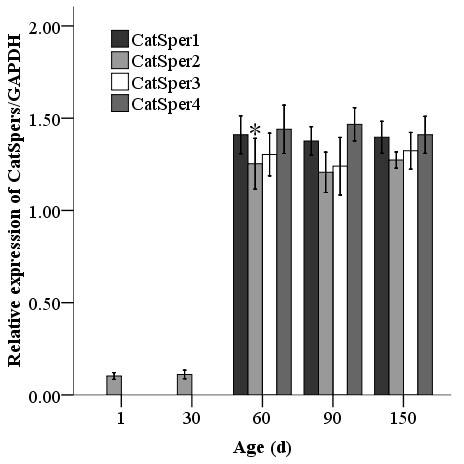
**Temporal expression of porcine CatSpers in testes**. Relative expression of CatSper1-4/GAPDH in testes at days 1 (n = 5), 30 (n = 4), 60 (n = 3), 90 (n = 3), and 150 (n = 3) of age. * indicates significant change compared to the former time point (P < 0.05).

The temporal expression profile of CatSper mRNAs during testicular development was determined by quantitative real-time RT-PCR. Their expression levels at different stage of development after birth from day 1 to 150 of age were summarized in Figure 7. At Days 1 and 30, CatSper1, CatSper3 and CatSper4 were not detectable, while Catsper2 began to be transcribed and was detected at a low level by Day 1. This low expression level was maintained until Day 30; whereupon, the expression of this gene transcript increased dramatically from Days 30 to 60 (Figure 7). After Day 60, all CatSper genes were abundantly expressed, and maintained at seemingly high constitutive level without alteration until Day 150 (Figure 7), which is the last age that was analyzed.

## Discussion

The CatSper proteins 1-4 form heterotetrameric, pH, and voltage dependent Ca^2+ ^permeable ion channels that are essential for hyperactivated motility and required for male fertility in mice [1,3,6,8,28]. CatSpers1-4-null mice are infertile and the spermatozoa fail to develop hyperactivated motility. These proteins have been studied extensively for their functionality and roles in reproduction in the mouse and human. However, no information is available in the pig. In the present study, we reported the full-length sequence of the four porcine CatSper cDNAs and deduced the amino acid sequences based on their gene sequences and further analyzed for their presumptive protein structure. The expression of these genes in different organs, in the male and female genital tracts, and in ejaculated sperm was investigated by qualitative RT-PCR. Furthermore, their gene expression patterns in the testes were studied by quantitative real-time RT-PCR from birth till sexual maturity (day 150 of age) as well. To our knowledge, this is the first report in pigs to show the spatial and temporal expression of porcine CatSper genes.

The CatSpers, belonging to the members of the voltage-gated ion channel superfamily [29], contain four alpha subunits (CatSper1-4) [30], and two auxiliary subunits, CatSperβ and CatSperγ [4,5]. In the current study, 24 CatSper protein sequences of four alpha subunits (CatSper1-4) from cattle, dog, human, mouse, pig and rat were analyzed and the transmembrane motifs were predicted by tmap program. As common protein domains found in the ion pore-forming subunits of this channel superfamily [2,9,26], six transmembrane regions denoted S1-S6 and a membrane re-entrant loop between S5 and S6 that forms part of the ion conducting pore were predicted. High species sequence identity and similar conserved pattern across the transmembrane and pore forming domains were revealed by multi-alignment. These data suggest the functional importance of these domains in regulation of sperm mobility and fertility.

Coiled-coils, a structural motif in proteins, in which 2-7 alpha-helices are coiled together like the strands of a rope[31], are well characterized as potential protein-protein interaction domains [32,33]. Such areas have also been found in multi-protein complexes such as the SNARE complex [27]. All four of the porcine CatSpers was predicted to contain a putative protein interaction motif near the carboxyl terminus in the current study. As discussed previously for mouse and human CatSpers [2,11,13], these regions could play important roles in the formation of CatSper complex. However, experiments are needed to address the roles of the putative CatSper protein interaction motifs have not been reported.

The spatial expression of CatSpers in different organs has been studied in the mouse and human. In both species, CatSper1, 2 and 3 are exclusively expressed in the testes, not in other organs [1,3,11,13]. In mice, CatSper 4 is only expressed in the testes not in other organs [11]. However, in humans, CatSper4 is detected in the testes, placenta and lung as well [2]. Based on their restricted expression patterns and their vital roles in sperm functions, CatSper family members are predicted to be potential targets for male infertility screening and ideal targets for contraception [1,13,15]. In the current study, CatSper2 and 4 were found to be expressed in the testes of boars among 26 organs studied, while the expression of porcine CatSper1 was detected in both testes and hypothalamus organs and CatSper3 was distributed extensively in the nervous organs (brain, cerebellum, pituitary, hypothalamus, hippocampus), and male reproductive tracts (testes and rete testes, caput epididymidis, prostate and seminal vesicle). The pattern of CatSper3 in porcine organs contrasted with that of mouse, which is specifically expressed in testes [3,11], which may be explained by evolutionary divergence of gene function across species. However, the function of this gene in the various porcine organs is uncertain.

Expression of mouse CatSpers is thought to be androgen dependent [14,15]. CatSpers are first detected after the first round of meiosis, which generally occurs at about 15 days of age, and sharply up-regulated at day 21, which coincides with the appearance of round spermatids in the developing testes, and peak around Day 42, when sexual maturity is reached in the mouse [34]. In the present study, CatSper mRNAs only minimally expressed at Days 1 and 30 of age. These transcripts were highly expressed by Day 60, when round spermatids become evident in the cross sections of seminiferous tubules in the testes of Meishan boars, suggesting that these germ cells are the primary source for this gene transcript [17], These findings are in agreement with a previous mouse study [14]. However, these forms maintain an expression level without a precipitous increase between Days 60 to 150, even though boars reached sexual maturity at 120 days of age [17]. This pattern differs from the temporal expression profile of mouse CatSpers, which rise during this comparable developmental time [14,15]. Perhaps, sexual maturity in male Meishan boars is less dependent on these Catsper proteins than male mice.

In agreement with observations of the mice [14], the expression of the CatSper2 gene in the current study commenced at an earlier developmental stage (Day 1) than other CatSper genes. These differences between the temporal expression profiles of the CatSper transcripts in porcine testis development suggest different mechanisms govern the expression of these various transcripts. Additionally, developmental changes in the somatic and germ cell population of the testes might drive these alterations [35]. It is also possible that there is fine orchestration and function of the CatSper2 and others forms at different stages of testis development. In CatSper2 null mice, the males are infertile because while spermatozoa are produced, they are not capable of forward propulsion and acquiring hyperactivated motility, necessary for successful ability to navigate within the female reproductive system and contact ovulated oocytes within the oviduct [6,8,13]. The rapid expression of CatSper2 compared to other forms and transgenic mice studies highlights that CatSper2 might be the most critical for normal male reproduction.

In the current study, low expression levels of CatSper3 and CatSper4 were detected in ejaculated sperm; whereas CatSper1 and CatSper2 were not detectable even after 42 PCR cycles. The origin of sperm RNAs remains unclear. Several RNA populations in ejaculated sperm of mammals [36-39], including the transcripts encoding for some ion channels have been reported [40]. Since the protein translation of sperm RNA has been demonstrated by Gur and Breitbart [41], the transcripts in sperm could play an essential role for sperm functions, such as motility, capacitation, and acrosome reaction. Furthermore, the level of CatSper transcripts in human ejaculated sperm has been positively correlated with overall sperm motility [14,16]. Further studies are needed to investigate the biological role of CatSper RNAs in porcine sperm.

While it would have strengthened these findings to localize and verify the protein expression pattern of these genes in the testes, this proved problematic for several reasons. We attempted immunohistochemistry to detect their presence of proteins by using several antibodies from Santa Cruz Biotechnology such as CatSper1 (H-20, sc-21181), CatSper2 (H-60, sc-98539), CatSper3 (H-216, sc-98702); and CatSper4 (T-17, sc-83126). However, specific location was not possible in in the testes, which is presumably due to the lack of cross reactivity for these antibodies that were developed against the protein forms for other species, and in some cases against non-conserved regions within these proteins. Additionally, we performed Western Blotting with CatSper antibodies that we developed against the porcine forms. The antibody against CatSper2 seemingly worked but several additional bands besides the expected size band of 62 kDa were correspondingly observed. We are currently sorting out whether these are additional bands represent isoforms of CatSper2 or are non-specific. We are further optimizing these protein expression procedures to determine whether they match the mRNA expression profile.

## Conclusions

In summary, we have cloned the full length cDNAs of porcine CatSper1, CatSper2, CatSper3, and CatSper4, and characterized the spatial and temporal expression profiles in the testes and other organs. CatSper2 and 4 were found to be testis-specific; whereas CatSper1 expression was confined to the testes and hypothalamus. However CatSper3 was distributed extensively across the nervous organs and male reproductive organs. The transcripts of CatSper3 and CatSper4 were identified in mature spermatozoa. Substantial expression of CatSper genes was observed in the testes at 60 days of age and with high expression levels persisting through 150 days of age. This study provides essential molecular data that can be employed in follow-up studies that will address the biological roles of CatSpers in normal porcine male reproductive function.

## Competing interests

The authors declare that they have no competing interests.

## Authors' contributions

CS, BG, HW and XW participated in the design of the study, collected the materials, carried out all experiments and CS, GC and JM drafted the manuscript. CS, BG, HW, XW, YX and BL collected the materials and helped to carry out RT-PCR, RACE. All authors read and approved the final manuscript.
